# NR0B2 Is a Key Factor for Gastric Diseases: A GEO Database Analysis Combined with Drug-Target Mendelian Randomization

**DOI:** 10.3390/genes15091210

**Published:** 2024-09-16

**Authors:** Zhengwen Li, Lijia Xu, Dongliang Huang, Chujie Li, Guido R. M. M. Haenen, Ming Zhang

**Affiliations:** 1 School of Pharmacy, Chengdu University, 2025 Chengluo Avenue, Chengdu 610106, China; xulijia@cdu.edu.cn (L.X.); huangdoliang@gmail.com (D.H.); 2 Precision Medicine, Faculty of Health, Medicine and Life Sciences, Maastricht University, 6200 MD Maastricht, The Netherlands; c.li@maastrichtuniversity.nl; 3 Department of Pharmacology and Personalized Medicine, Faculty of Health, Medicine and Life Sciences, Maastricht University, 6200 MD Maastricht, The Netherlands; g.haenen@maastrichtuniversity.nl; 4 College of Food Science and Engineering, Hainan University, 58 Renmin Road, Haikou 570228, China

**Keywords:** *NR0B2*, GEO, gastric diseases, drug-target Mendelian randomization

## Abstract

Small Heterodimer Partner (SHP; *NR0B2*) is an orphan receptor that acts as a transcriptional regulator, controlling various metabolic processes, and is a potential therapeutic target for cancer. Examining the correlation between the expression of *NR0B2* and the risk of gastric diseases could open a new path for treatment and drug development. The Gene Expression Omnibus (GEO) database was utilized to explore *NR0B2* gene expression profiles in gastric diseases. Co-expressed genes were identified through Weighted Correlation Network Analysis (WGCNA), and GO enrichment was performed to identify potential pathways. The Xcell method was employed to analyze immune infiltration relationships. To determine the potential causal relationship between *NR0B2* expression and gastric diseases, we identified six single-nucleotide polymorphisms (SNPs) as a proxy for *NR0B2* expression located within 100 kilobases of *NR0B2* and which are associated with triglyceride homeostasis and performed drug-target Mendelian randomization (MR). Bioinformatics analysis revealed that *NR0B2* expression levels were reduced in gastric cancer and increased in gastritis. GO analysis and Gene Set Enrichment Analysis (GSEA) showed that *NR0B2* is widely involved in oxidation-related processes. Immune infiltration analyses found that *NR0B2* was associated with Treg. Prognostic analyses showed that a low expression of *NR0B2* is a risk factor for the poor prognoses of gastric cancer. MR analyses revealed that *NR0B2* expression is associated with a risk of gastric diseases (*NR0B2* vs. gastric cancer, *p* = 0.006, OR: 0.073, 95%CI: 0.011–0.478; *NR0B2* vs. gastric ulcer, *p* = 0.03, OR: 0.991, 95%CI: 0.984–0.999; *NR0B2* vs. other gastritis, *p* = 0.006, OR:3.82, 95%CI: 1.468–9.942). Our study confirms the causal relationship between the expression of *NR0B2* and the risk of gastric diseases, and highlights its role in the progression of gastric cancer. The present study opens new avenues for exploring the potential of drugs that either activate or inhibit the *NR0B2* receptor in the treatment of gastric diseases.

## 1. Introduction

NR0B2 (Nuclear Receptor subfamily 0 group B member 2), also known as Small Heterodimer Partner (SHP), is a member of the nuclear receptor family. It primarily functions as a transcriptional repressor by interacting with other nuclear receptors and small molecule drugs, regulating numerous metabolic pathways [1]. These pathways include the homeostasis of glucose, bile acids, cholesterol, triglycerides, and fatty acids in various organs [2].

The role of *NR0B2* gene has been explored in several cancers. Zhu et al. [3], found that a higher *NR0B2* expression is significantly associated with longer recurrence-free survival and progression-free survival in liver cancer, as determined through the analysis of public databases. In breast tumors, *NR0B2* expression is negatively correlated with *FOXP3*, a marker for Tregs, suggesting that *NR0B2* may reduce immunosuppression in the tumor microenvironment [4,5]. Similarly, Prestin et al. [6] revealed that the downregulation of *NR0B2* in renal cancer is associated with cancer development and progression. They speculated that *NR0B2* triggers G2 arrest in renal cancer cells, preventing them from entering mitosis. Further research suggests that the hypermethylation of the *NR0B2* promoter region is the primary mechanism behind its downregulation [7]. The growth-inhibiting effect of SHP (*NR0B2*) might also be mediated by its targeting of NF-κB, P53, C-JUN, HDAC6, NUR77, and BCL2, all of which are involved in apoptosis and cell cycle progression [8]. Remarkably, there are only a few reports on the role of *NR0B2* in gastric diseases.

Due to dietary factors, the incidence of gastric diseases in China is relatively high, posing a significant threat to public health [9,10]. The National Cancer Center in China reported that, in 2022, the incidence of gastric cancer was 35.87 per 100,000 people, with a mortality rate of 26.04 per 100,000 people, ranking it fifth and third in terms of the incidence and mortality of malignant tumors, respectively. The 5-year relative survival rate is approximately 40.5%. Molecular markers such as EGFR, p53, and HER2 are commonly overexpressed and activated in gastric cancer [11], and drug treatments targeting these genes are gradually entering clinical trials. However, the heterogeneity of gastric cancers, their complex tumor microenvironment, and their high impact on patients’ lives necessitate tailored therapies. Therefore, efforts to identify new molecular markers and develop new drugs must continue. SHP (*NR0B2*) is one of these potential targets.

As already stated above, SHP (*NR0B2*) is a nuclear receptor. Nuclear receptors play crucial roles in numerous physiological processes, including growth, metabolism, and differentiation. In the global pharmaceutical market, nuclear receptor ligands constitute about 15% to 20% of small-molecule drugs [12,13,14]. Some well-known examples are tamoxifen, raloxifene, genistein, diethylstilbestrol, and equine estrogens, which are used to treat conditions like menopausal symptoms, osteoporosis, and breast cancer. However, the majority of these drugs target orphan receptors, xenobiotic receptors, classic RXR heterodimer receptors, and classic steroid receptors. NR0B2-targeting drugs are relatively rare.

Recent research has shown that 5-(diethylamino-sulfonyl)-3-hydroxy-2-naphthoic acid (DSHN) can activate NR0B2, inhibiting the p65 activation of the CCL2 promoter. This inhibition leads to the reduced expression and secretion of CCL2, consequently suppressing the migration and invasion of hepatocellular carcinoma (HCC) cells [15]. These findings exemplify the therapeutic potential of SHP (*NR0B2*) in liver treatment. However, it is not yet known if this also applies to gastric diseases. To explore the correlation and causation between *NR0B2* expression and gastric diseases, we plan to combine a GEO database analysis with drug-target Mendelian randomization. GEO is currently the largest, fully public gene expression resource established by the National Institutes of Health (NIH), containing more than 4000 datasets and over 7,000,000 samples at present, which enable researchers to freely download and perform bioinformatics analyses [16] and discover novel tumor biomarkers. By analyzing public databases, we can explore the expression differences of *NR0B2* in the stomach and its physiological and pathological roles. However, the results of GEO analyses are often blurred by confounding factors, leading to potential biases [17]. Often, the relationships found are associations rather than causal relationships. Mendelian randomization (MR) has emerged as a relatively reliable method for finding causal relationships. This method employs single-nucleotide polymorphisms (SNPs) as instrumental variables (IVs) to infer the causal relationships between exposure factors and outcomes [18].

The Mendelian randomization (MR) method is based on Mendel’s law, where alleles of single-nucleotide polymorphisms (SNPs) segregate and are randomly inherited from parents to offspring. This process is similar to the random treatment assignment in randomized controlled trials (RCTs) [19]. In the MR method, “exposure” refers to a continuous or dichotomous risk factor for a disease, while “outcome” refers to the disease itself or disease-related traits. This method is widely used to estimate the causal effects of various diseases [20,21,22,23]. Additionally, when the genetic variation is related to the target gene of a drug-associated protein, this approach is known as drug-target Mendelian randomization [24]. A notable example is the lipid-lowering drug target gene PCSK9. Researchers have identified genetic variants associated with PCSK9 and low-density lipoprotein (LDL) levels, using them to evaluate the relationship between lipid-lowering drugs and various diseases [25,26]. Therefore, we used MR to explore the causal relationship between *NR0B2* expression and gastric diseases. Summary-level data from genome-wide association studies (GWAS) in public databases were used to uncover these relationships. The causal relationships found might inspire new insights into the development of new drugs, or expand the use of existing drugs.

## 2. Materials and Methods

### 2.1. Data Source and Preprocessing

Gene chip datasets for gastric diseases were obtained from the GEO database, including GSE138631 (19 control, 71 gastric cancer samples), GSE26942 (12 control, 205 gastric cancer samples), GSE179252 (38 control, 38 gastric cancer samples), GSE236522 (33 gastric cancer samples), GSE55696 (19 chronic gastritis samples), GSE130823 (47 gastritis samples), and GSE233973 (9 control, 27 gastritis samples). We combined GSE138631 and GSE26942 due to the similarity of their source data. For the MR analysis, gastric diseases were set as the outcomes, and their related GWAS summary data were downloaded from https://gwas.mrcieu.ac.uk/(accessed on 8 July 2024). The GWAS data used in the study are shown in Table 1 below.

### 2.2. Bioinformatics Analysis

We conducted a weighted gene co-expression network analysis of the obtained GEO datasets, selecting genes based on their proximity to *NR0B2* and merging them. This process yielded 7 co-expressed genes for gastric cancer and 58 co-expressed genes for gastritis. Subsequently, the identified genes were subjected to a Gene Ontology (GO) functional enrichment analysis using the “clusterProfiler” and “enrichplot” packages. The normalization criteria were set to *p* < 0.05 and q < 0.05. The GO terms include Biological Processes (BP), Molecular Functions (MF), and Cellular Components (CC). The results were ranked based on the enrichment ranking thresholds (e.g., *p*-values). A Gene Set Enrichment Analysis (GESA) was performed to confirm the pathway alignment of the GO results. The Xcell algorithm [27] was used to assess the relationship between *NR0B2* expression and immune cell infiltration. Additionally, the association between *NR0B2*’s expression and prognosis was examined using the GSE236522 dataset. The results were visualized using “ggplot2”.

### 2.3. Mendelian Randomization Analysis

To determine *NR0B2* expression, we used single-nucleotide polymorphisms (SNPs) located within 100 kilobases of *NR0B2* that are associated with triglyceride homeostasis, which were sourced from the UK Biobank GWAS (SNPs = 12,321,875). A genome-wide significance *p* < 5 × 10^–8^ was used as the measurement instrument and clumped them at linkage disequilibrium R2 < 0.3. This resulted in the identification of 6 moderately correlated SNPs.

Five MR analysis methods were used: MR-Egger, weighted median, inverse-variance weighted (IVW), simple mode, and weighted mode. Sensitivity analyses, including a heterogeneity analysis and pleiotropy testing, were performed using Cochran’s Q test and the MR-Egger method, where *p* > 0.05 indicated no heterogeneity. In cases of heterogeneity, the random-effect model was used to replace the IVW results. A leave-one-out analysis was performed to evaluate the influence of each SNP on the outcome variable by sequentially removing each SNP. Funnel plots and scatter plots were made to visually assess the heterogeneity and pleiotropy of the MR results. The total F value of the IVs was calculated. When the F value of the IVs exceeded 10, the IVs were considered to be reliable. The IVs used are listed in Table 2.

## 3. Results

### 3.1. NR0B2’s Down-Regulation in Gastric Cancer and Up-Regulation in Gastritis

**Figure 1:**
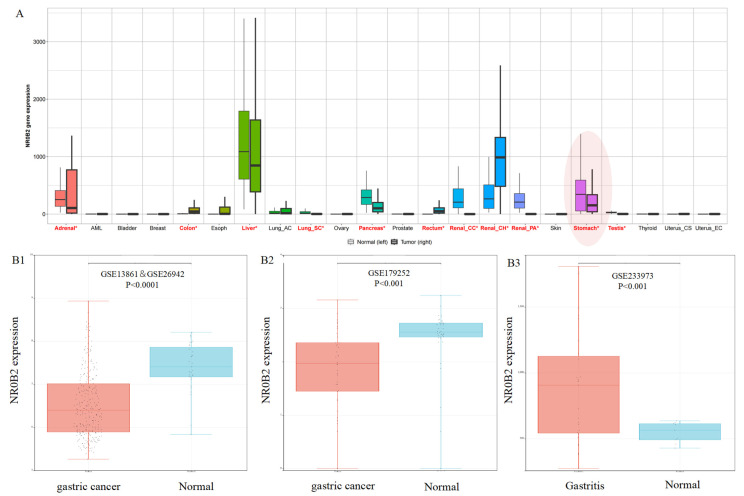
*NR0B2* expression in various human tumors (**A**), gastric tumors (**B1**,**B2**), and gastritis (**B3**). The words in red with an * represent *p* < 0.05 in panel (**A**).

We assessed *NR0B2* expression using data from the TNMplot website [28], GSE138631&GSE26942 and GSE179252 for gastric cancer, and GSE233973 for gastritis. The datasets GSE236522, GSE130823, and GSE55696 were excluded due to the lack of a control group. As shown in Figure 1A, *NR0B2* expression in both normal and tumor tissues of the kidney, liver, and stomach is higher than that in other organs. Additionally, Figure 1A shows that *NR0B2* expression in stomach tumors is significantly lower than that in normal stomach tissue. The latter aligns with the results from the GSE138631&GSE26942 (*p* < 0.0001) and GSE17952 (*p* < 0.001) datasets (Figure 1(B1,B2)). Interestingly, as shown in Figure 1(B3), *NR0B2* expression is higher in gastritis samples compared to the control (*p* < 0.001). This suggests that *NR0B2* plays different roles in gastric cancer and gastritis.

### 3.2. NR0B2 Is Important for Lipid Metabolism and Oxidation-Related Processes

**Figure 2:**
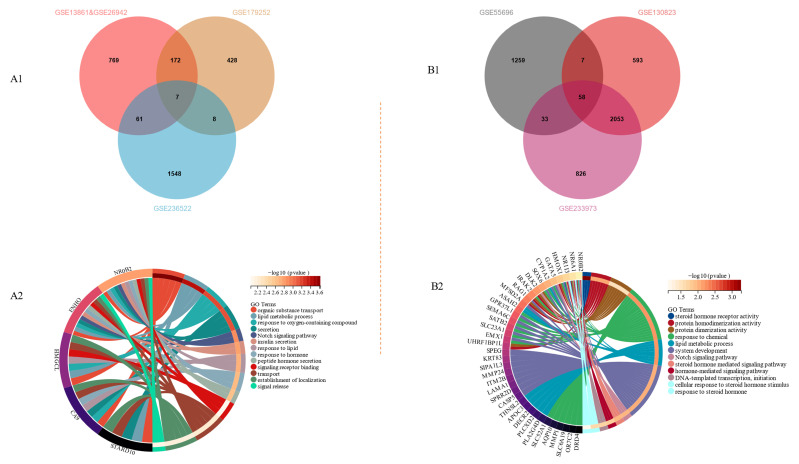
Venn diagram of co-expression genes generated by WGCNA for gastric cancer (**A1**) and gastritis (**B1**); GO enrichment analyses of gastric cancer (**A2**) and gastritis (**B2**), FDR < 0.25 and *p* < 0.05. Enrichment results with only one gene were not selected.

We explored the biological function of *NR0B2* in gastric diseases through WGCNA, identifying 7 co-expression genes in gastric cancer (Figure 2(A1)) and 58 co-expression genes in gastritis (Figure 2(B1)). The merged genes can be found in Table S1. The GO enrichment analysis showed that *NR0B2* and its co-expression genes are involved in various processes in gastric cancer (Figure 2(A2)) and gastritis (Figure 2(B2)), including organic substance transport and the Notch signaling pathway. Although there was no overlap between the merged genes in gastric cancer and gastritis (except for *NR0B2*), the GO results suggest that *NR0B2* and its co-expressed genes participate in lipid metabolic and oxidation-related processes in gastric cancer and gastritis. It also reveals the importance of inflammation and antioxidant adjustment, which is consistent with the results from the GESA (see Supplementary Figure S3); the Forest Plot of its GO enrichment can be seen in Supplementary Figure S4.

### 3.3. NR0B2 Is Associated with Several Immune Infiltrations

**Figure 3:**
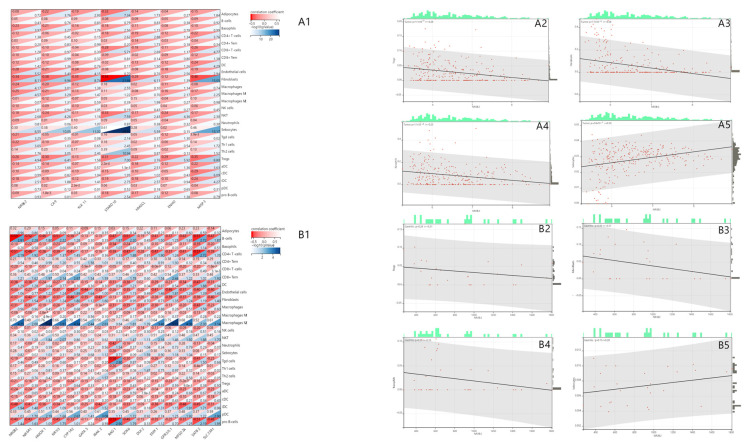
Correlation analysis between *NR0B2* expression and tumor-infiltrating immune cells in gastric cancer (panels (**A**)) and gastritis (panels (**B**)). (**A1**) A heatmap of the relationship between 7 genes and 26 immune-related cells, where the red bar and blue bar represent the correlation coefficient of gene expressions with immune-related cells and the corresponding −log10 (*p* Value), respectively. (**A2**) *NR0B2* expression vs. Tregs; (**A3**) *NR0B2* expression vs. fibroblasts; (**A4**) *NR0B2* expressions vs. basophils; (**A5**) *NR0B2* expression vs. sebocytes. (**B1**) A heatmap of the relationship between 15 genes and 26 immune-related cells, in which genes with the highest association with *NR0B2* in the GO enrichment were selected; (**B2**) *NR0B2* expression vs. Tregs; (**B3**), *NR0B2* expression vs. fibroblasts; (**B4**) *NR0B2* expressions vs. basophils; (**B5**) *NR0B2* expression vs. sebocytes. The results are based on the GSE138631&GSE26942 (gastric cancer) and GSE233973 (gastritis) databases.

It has been reported that *NR0B2* expression affects the innate immune response in liver cancer, where a partial but significant correlation between lower *NR0B2* expression levels and a higher tumor infiltration of B cells and CD8+ T cells has been found [3]. In order to examine whether a similar relationship exists in gastric cancer, we determined the correlation between *NR0B2* expression and tumor-infiltrating lymphocytes using the Xcell database (Figure 3(A1)).

Our analysis showed no significant relationship between *NR0B2* expression and the infiltration of CD4+ or CD8+ cells in gastric cancer. However, we found a negative correlation with Tregs (*p* < 0.01, Figure 3(A2)) and fibroblasts (*p* < 0.001, Figure 3(A3)) that is consistent with previous reports [29]. Additionally, we observed a significant correlation between *NR0B2* expression and basophils (Figure 3(A4)), suggesting a role for *NR0B2* in the inflammatory process in gastric cancer. The strong correlation with sebocytes (*p* < 0.001, Figure 3(A5)) further supports *NR0B2*’s crucial role in lipid metabolism. In contrast, in gastritis, *NR0B2* expression did not correlate with CD4+ T cells, neutrophils, Tregs (Figure 3(B2)), fibroblasts (Figure 3(B3)), basophils (Figure 3(B4)), or sebocytes (Figure 3(B5)). However, we found a significant correlation with macrophages (*p* < 0.001). The differences in the correlations found in gastric cancer and gastritis further indicate that *NR0B2* plays different roles in these diseases.

### 3.4. MR Analysis Results Show That NR0B2 Has a Causal Relationship with Gastric Diseases

**Figure 4:**
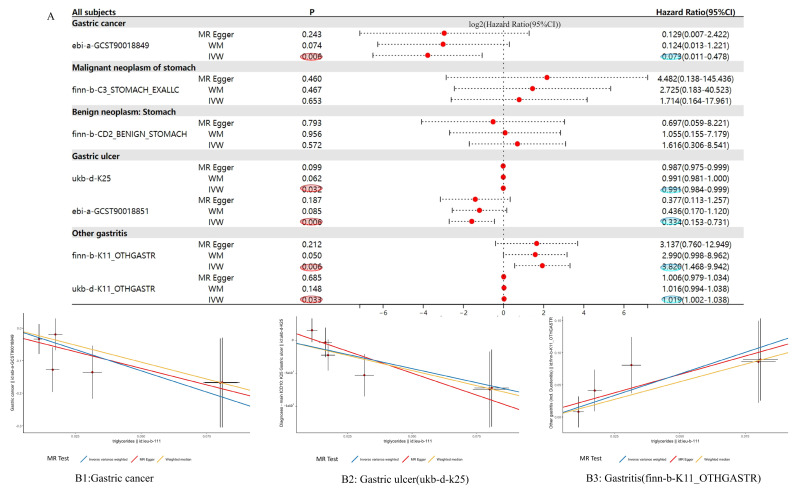
MR results of the *NR0B2* expression in several gastric diseases. The table in panel (**A**) shows the MR results for each gastric disease, with P representing the *p* value and the Hazard Ratio being equal to the OR. The *p* values of these causal relationships that are < 0.05 are marked in red. The *p*-values of OR that are < 0.05 are marked in blue. The panels (**B1**–**B3**) depict the scatter plots of *NR0B2* vs. gastric cancer, gastric ulcer, and gastritis, respectively.

To establish causality between *NR0B2* expression and gastric diseases, we conducted an MR analysis. As displayed in Figure 4(B1), *NR0B2* expression was found to have a causal relationship with gastric cancer (*p* = 0.006; OR = 0.073), indicating that a higher expression of *NR0B2* protects against gastric cancer. No correlation with benign tumors was found. A similar relationship between the expression of *NR0B2* and gastric ulcers was found in the ukb-d-K25 dataset (*p* = 0.032; OR = 0.991) and the ebi-a-GCST90018851 dataset (*p* = 0.006). Conversely, Figure 4(B3) shows that, for gastritis, a higher expression of *NR0B2* is related to a higher risk of the disease, similar to the relationship found in the fin-b-k11 (*p* = 0.033) and ukb-d-k11 (*p* = 0.006) cohorts. These findings again suggest that *NR0B2* plays different roles in gastric cancer and gastritis.

### 3.5. Heterogeneity Analysis Results of MR

**Figure 5:**
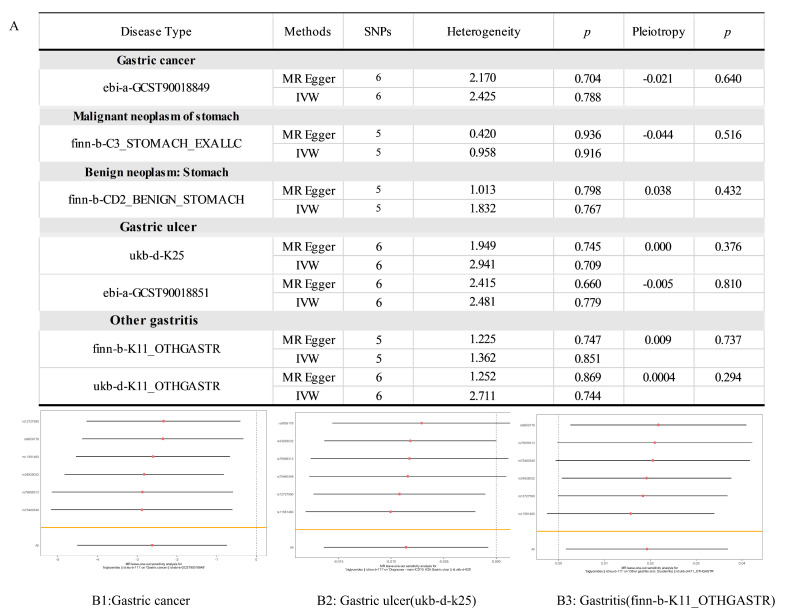
Heterogeneity and pleiotropy analysis of gastric diseases: (**A**) heterogeneity and pleiotropy results; (**B1**–**B3**) Forest Plots of leave-one-out tests for gastric cancer, gastric ulcers (ukd-d-k25), and gastritis (finn-b-K11_OTHGASTR).

The heterogeneity analysis (Figure 5A) indicated no evidence of heterogeneity in positive groups, suggesting that the results were reliable. The leave-one-out sensitivity analysis (Figure 5(B1–B3)) showed that no SNP significantly biased the outcome for gastric cancer, gastric ulcer, or gastritis. The results for benign gastric cancer and gastric ulcers are shown in Supplementary Figures S6 and S7.

## 4. Discussion

Our analysis aligns with previous findings, demonstrating that *NR0B2* expression is reduced in various cancers compared to normal tissue. Zhu et al. [3] found a significant decrease in *NR0B2* expression across over 30 datasets in the Oncomine database, including gastric cancer. Our analysis of the datasets GGSE138631, GSE26942, and GSE179252 corroborates this finding. Although our data from the TCGA database did not show this trend, results from the pan-cancer analysis using TNMplot confirm significant differences in *NR0B2* expression in gastric cancer. This consistency across multiple databases reinforces the conclusion that *NR0B2* expression is diminished in gastric cancer.

Regarding the mechanisms involved, in vitro studies suggest that the overexpression of *NR0B2* can inhibit hepatocellular carcinoma (HCC) lesions’ formation and tumor growth, potentially through SHP’s (the protein corresponding to *NR0B2*) regulation of cyclin D1 [8]. Furthermore, SHP’s interaction with the p53 and Mdm2 proteins influences their ubiquitination and stability, underscoring SHP’s role as a tumor suppressor in HCC. However, the relevance of these mechanisms to gastric diseases requires further investigation. The transcriptome study of gastric cancer patients by Adriana Carino et al. [30] highlighted that *NR0B2*, alongside factors like *CCL19*, *PTGS2*, and *FN1*, reduces inflammation—a known cancer risk factor. The anti-inflammatory role of SHP (*NR0B2*) might involve the inhibition of NF-κB p65, preventing the CCL2-mediated recruitment of monocytes, memory T cells, and dendritic cells to tumors.

The GO analysis revealed that *NR0B2* and its co-expressed genes in gastric cancer are predominantly involved in lipid metabolism and oxidation reactions. Notably, genes like *CA9*, *ENHO*, *HMGCL*, *NR0B2*, and *STARD10* are linked to inflammatory cell recruitment. In gastritis, the co-expressed genes *NR1I3*, *NR6A1*, and *NR0B2* are associated with anti-inflammatory steroid hormone synthesis and metabolism. These findings further support inflammation’s role in both diseases. Due to a lack of data, similar analyses could not be performed for gastric ulcers.

Our GESA (Figure S3) shows *NR0B2*′s involvement in the NRF2 antioxidant pathway in both gastric cancer and gastritis. NRF2 can activate PPARγ and NR0B2, reducing fatty acid production and competing with the cellular antioxidant system. Recent studies also suggest that SHP (*NR0B2*) regulates myeloid cell inflammasomes, reducing IL-1β secretion and subsequent Treg expansion, which is linked to cholesterol homeostasis [31]. This is consistent with our immune infiltration analysis, where Tregs, which are crucial for maintaining self-tolerance and avoiding excessive immune responses, are associated with *NR0B2* expression. Interestingly, in gastritis, *NR0B2* expression significantly correlates with M2 macrophage infiltration. M2 macrophages are key in IL-10 and TGF-β secretion, contributing to inflammation. This observation aligns with previous findings of *NR0B2*’s association with a favorable cancer prognosis, as noted in GSE197252 (Figure S5).

In order to determine the causal relationship of *NR0B2* in gastric diseases, we conducted a drug-target Mendelian randomization analysis. Drug-target Mendelian randomization has certain requirements for selecting SNPs [32]. Considering that *NR0B2* is involved in the regulation of the metabolism of triglycerides, we extracted SNPs in genes that are related to the exposure of triglycerides. We explored the relationship between these SNPs and gastric cancer, gastric ulcers, and gastritis. No heterogeneity was found in our study. Some studies have questioned the relationship between triglycerides and digestive system cancers, since their findings did not support this relationship [33]. A possible explanation is that triglyceride content is influenced by many factors, causing the effects of *NR0B2* to be eclipsed by these other factors. In addition, Yan et al. [34] reported that high levels of cholesterol are a risk factor for gastric cancer through the IVW method. Given that *NR0B2* also plays a key role in cholesterol regulation, we tried to extract variables related to *NR0B2* from cholesterol and conducted a two-sample Mendelian randomization analysis of gastric cancer. However, our analysis did not show a significant correlation. This indicates that although cholesterol might have an impact on gastric cancer, it is unlikely to be regulated by *NR0B2*.

An intriguing finding of the present study is that elevated *NR0B2* is a risk factor for gastritis. Studies have shown that inflammation and lipid metabolism are interrelated at multiple levels. For example, lipid metabolism disorders will affect the drifting of monocytes to M1/M2, which can induce inflammatory responses [35,36]. Some studies have also found that lipid metabolism disorders will also affect the Nucleotide-binding oligomerization domain (NOD)-like receptor (NLR) family pyrin domain containing 3 (NLRP-3), thus activating Caspase-1, converting pro IL-1β into mature IL-1β and thereby causing a strong inflammatory response [37]. Research indicates that the balance between orphan nuclear receptor Nr5a2 and its co-repressor SHP (*NR0B2*) likely determines the dysregulation of AP-1 in the pancreas, where AP-1 is pro-inflammatory [38]. Whether this mechanism exists in gastric diseases remains to be verified. Additionally, some studies have shown that the activation of the FXR pathway, an upstream regulator of SHP (*NR0B2*), can protect the gastric mucosa from inflammation-induced damage [39]. Whether this protective effect is mediated by SHP (*NR0B2*) also remains to be determined. The long-term high expression of *NR0B2* may affect the concentration of triglycerides, thereby causing lipid metabolism disorders, increasing the risk of gastritis. On the other hand, inflammatory factors could affect lipid metabolism through the NF-kB pathway or the PPAR pathway. Therefore, gastritis may cause lipid metabolism disorders. In order to maintain homeostasis, *NR0B2* levels are forced to increase.

In addition, in the Mendelian randomization analysis, several factors may invalidate the core assumptions of the Mendelian randomization (MR) model. These include weak instruments, the horizontal pleiotropy of genetic variations, linkage disequilibrium (LD), and population stratification. Pleiotropy occurs when a genetic variant influences the outcome through pathways other than the “genetic variant → exposure factor → outcome” relationship [19,23]. For studies using multiple genetic instrumental variables, various statistical methods, such as the MR-Egger method used in our research can detect pleiotropy. Moreover, when interpreting MR results, it is essential to link genetic variations to protein expression in order to provide a more biologically sound explanation. For instance, research has shown a complex causal relationship between pancreatic cancer and peripheral metabolites, where high-density lipoprotein (HDL) and very low-density lipoprotein (VLDL) were linked to pancreatic cancer through Mendelian randomization [40]. Given the influence of various proteins, such as ABCA1, PCSK9, and *NR0B2*, on lipid metabolism, it is also valuable to explore these proteins rather than solely focusing on the genetic susceptibility of these diseases.

While we observed that a decreased *NR0B2* expression is a risk factor for gastric cancer, it should be noted that in some individuals with gastric cancer, *NR0B2* expression is increased. This might be due to a different stage of the cancer or to a wrong classification. Also, there are many risk factors for gastric cancer, and *NR0B2* is only one of them. This can explain why *NR0B2* is not always decreased in gastric cancer. Another critical aspect is that although the increased expression of *NR0B2* has been found in GSE233973, the number of negative individuals is small, indicating the need to verify our result in a larger cohort.

## 5. Conclusions

Our study aims to analyze and explore the effect of *NR0B2* expression on gastric diseases using bioinformatics methods and drug-target Mendelian randomization methods. We found that the high expression of *NR0B2* is a favorable prognostic factor for gastric cancer and may reduce its incidence, potentially through Treg-mediated inflammatory regulation. In contrast, a high *NR0B2* level increases the risk of gastritis. *NR0B2* appears to be a potential target for drug development for gastric cancer, but we also need to be aware that such drugs may cause side effects such as gastritis.

## Figures and Tables

**Table 1:** Characteristics of the GWAS cohorts.

Characteristics	GWASID	Type	Sample Size	Number of SNPs
Triglyceride	ieu-b-111	Exposure	441,060	12,321,875
Gastric cancer	ebi-a-GCST90018849	Outcome	476,116	24,188,662
Malignant neoplasm of stomach	finn-b-C3_STOMACH_EXALLC	Outcome	174,639	16,380,305
Benign neoplasm: stomach	finn-b-CD2_BENIGN_STOMACH	Outcome	218,792	16,380,466
Gastric ulcer	ukb-d-K25	Outcome	361,194	10,452,088
Gastric ulcer	ebi-a-GCST90018851	Outcome	474,278	24,178,780
Other gastritis	finn-b-K11_OTHGASTR	Outcome	174,576	16,380,406
Other gastritis	ukb-d-K11_OTHGASTR	Outcome	361,194	13,356,120

**Table 2:** Final IVs generated from exposure associated with *NR0B2*.

IVs	Chromosome	Effect Allele	Other Allele	β	SE	*p*
rs12727590	1	G	A	0.016437	0.0025535	1.2 × 10^−10^
rs6659176	1	G	C	0.0314972	0.0036665	8.7 × 10^−18^
rs11581460	1	A	G	−0.011234	0.0020387	3.6 × 10^−8^
rs79598313	1	T	C	0.0800707	0.0065634	3.1 × 10^−34^
rs34928032	1	G	A	0.0174607	0.0026908	8.6 × 10^−11^
rs75460349	1	C	A	0.0808376	0.0066624	7 × 10^−34^

## Data Availability

All data used in this study are available in a public repository. The code involved in the data analysis process can be obtained by contacting the corresponding author.

## Supplementary Materials

The following supporting information can be downloaded at https://www.mdpi.com/article/10.3390/genes15091210/s1, Figure S1: WGCNA analysis of gastric cancer; Figure S2: WGCNA analysis of gastritis; Figure S3: Gene set enrichment analysis (GSEA); Figure S4: Forest Plot of GO enrichment; Figure S5: Gene prognostic analysis based on GSE179252; Figure S6: The leave-one-out analysis and scatter plot for the malignant neoplasm of stomach, benign neoplasm: Stomach, gastric ulcer, and Gastritis; Figure S7: Funnel plot for drug Mendelian randomization analyses of the causal effect of the expression of NR0B2 on gastric diseases; Table S1: The merged genes by WGCNA.
