# Supplementary material for: NR0B2 Is a Key Factor for Gastric Diseases: A GEO Database Analysis Combined with Drug-Target Mendelian Randomization
Source: Genes (Basel). 2024 Sep 16;15(9):1210. doi: 10.3390/genes15091210 (PMC11431353; doi:10.3390/genes15091210)
Supplement: Supplementary file 1 [file genes-15-01210-s001.zip › Supplementary.pdf]

genes

MDPI

Supplementary:

NR0B2 is a Key Factor for Gastric Diseases: GEO Database Analysis Combined with Drug-Target Mendelian Randomization

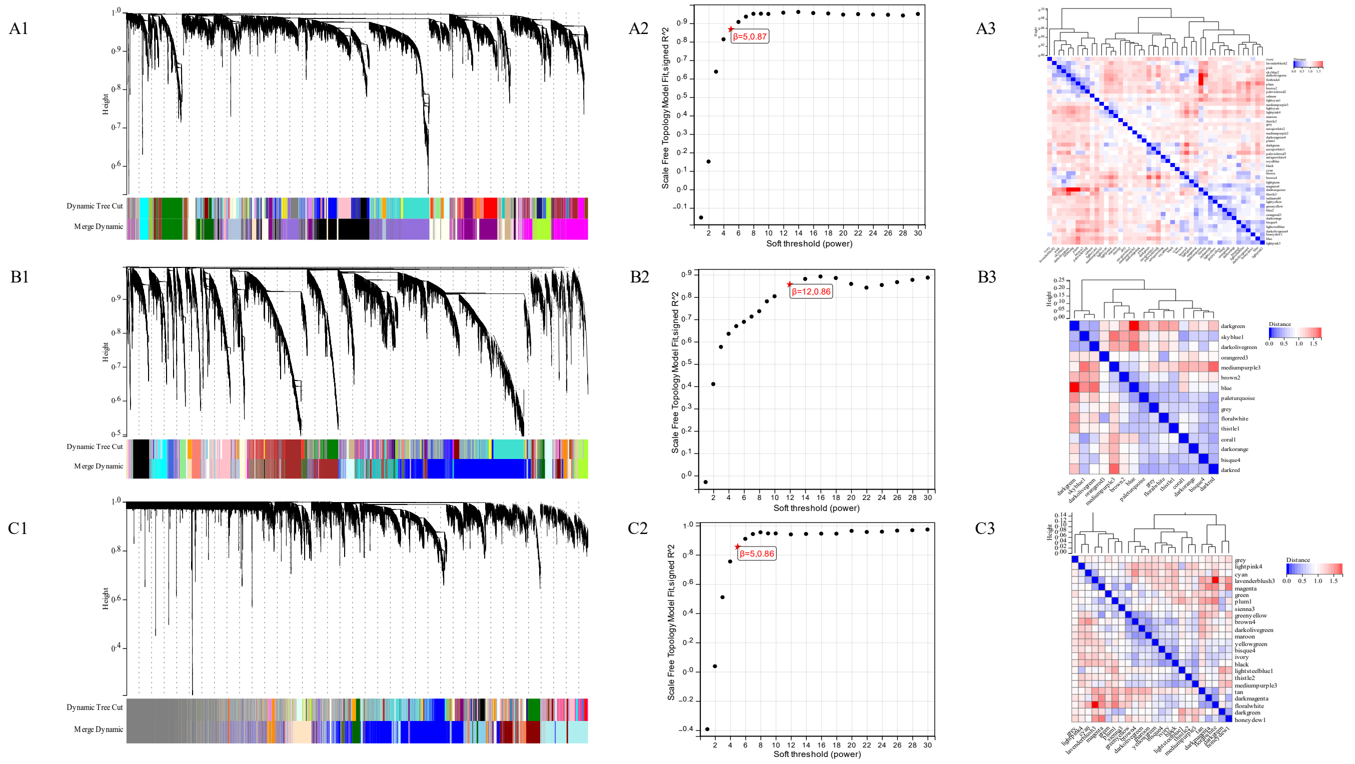

Figure. S1 WGCNA analysis. (A1) A clustering diagram of the gene modules denoted by distinct colors. The gene dendrogram was obtained by dissimilarity clustering, with the colors of the corresponding modules represented by colored lines, based on the consensus topological overlap. Each colored line represents a color-coded module containing a set of highly connected genes. (A2) The soft threshold selection (A3) The heatmap plot between modules. Where the A, B, C, based on GSE138631&GSE26942, GSE17952 and GSE236522, respectively.

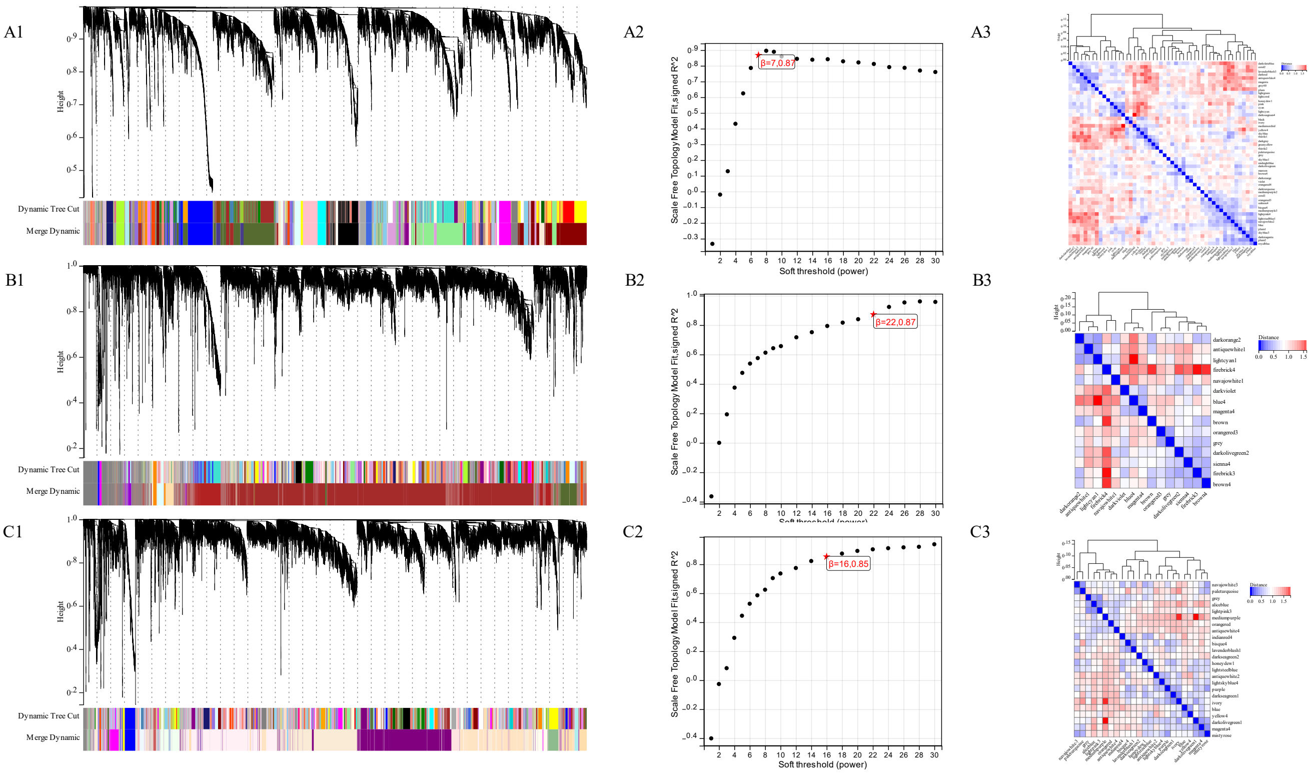

Figure. S2 WGCNA analysis. (A1) A clustering diagram of the gene modules denoted by distinct colors. (A2) The soft threshold selection (A3) The heatmap plot between modules. Where the A, B, C, based on GSE55696, GSE130823 and GSE233973, respectively.

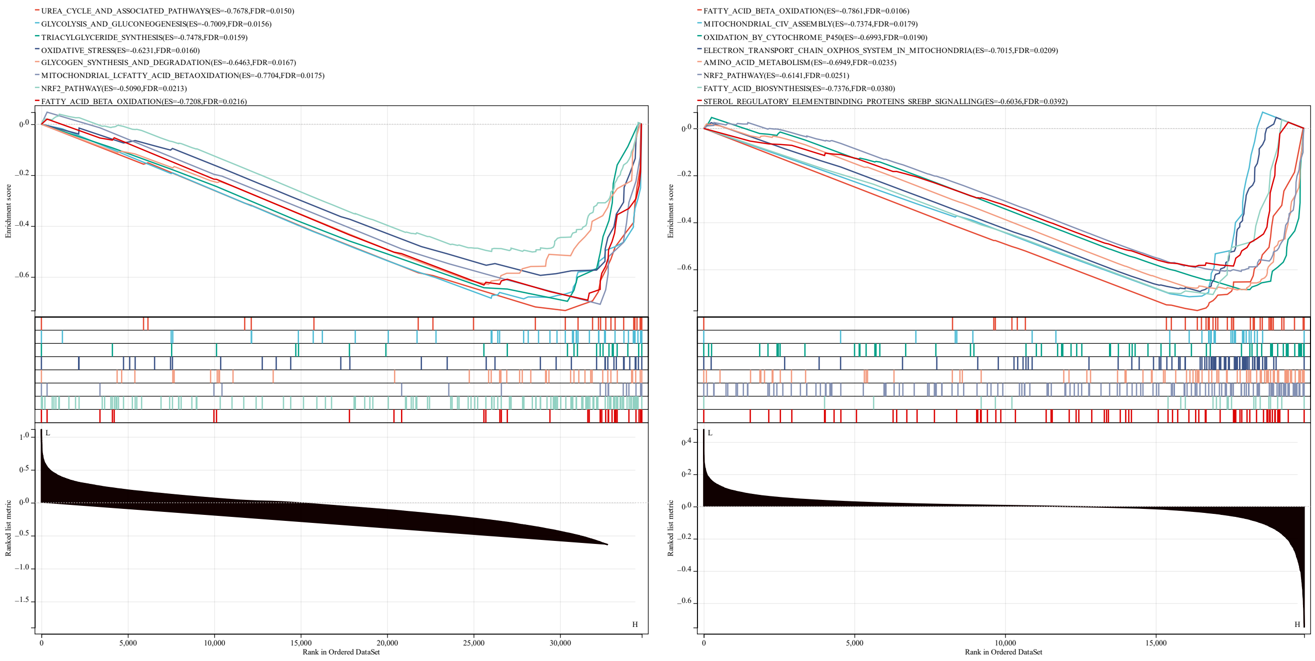

A B

Figure. S3 Gene set enrichment analysis (GSEA), according to the expression level of NR0B2, the samples has been divided into high expression group (>=50%) and low expression group (<50%), then performing analysis under wikipathways dataset, the minimum gene set to 5, the maximum gene set to 5000, P value of < 0.05 and a FDR of < 0.25 were considered statistically significant. Top 8 enrichment process has been listed. Results showed NR0B2 participated in Nrf2-pathway and fatty acid oxidation, both in gastric cancer(A) and gastritis(B), analysis based on GSE138631&GSE26942 and GSE233973.

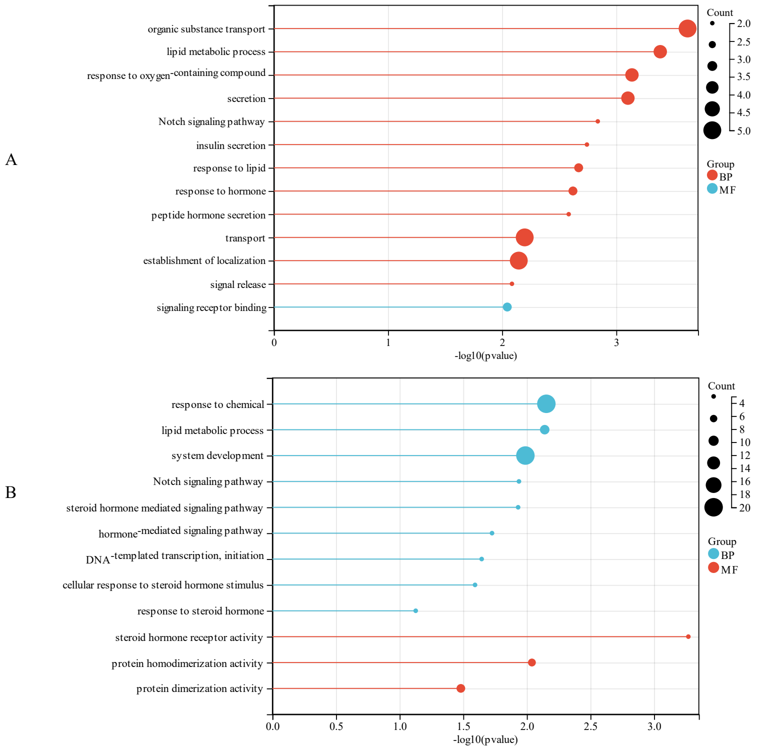

Figure. S4 Forest Plot of GO enrichment.

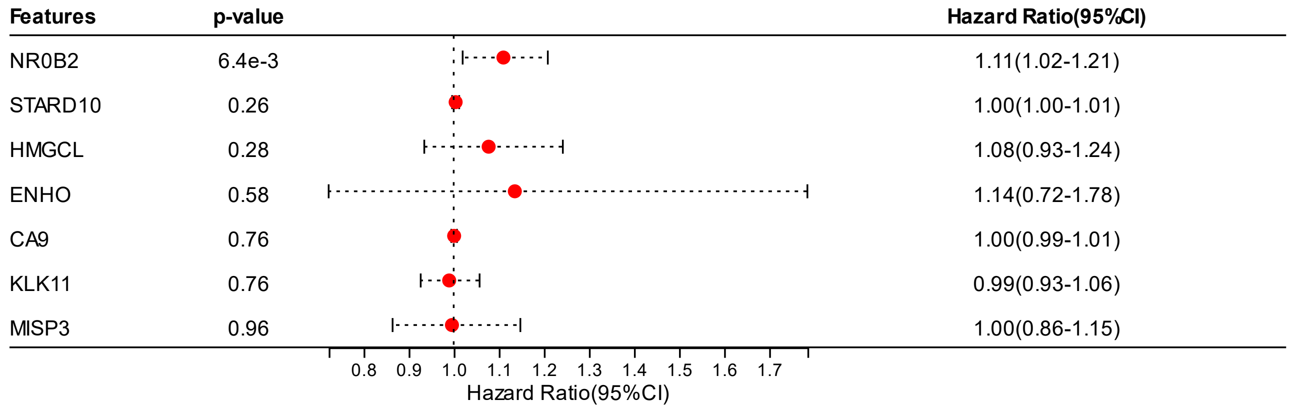

Figure. S5 Gene prognostic analysis based on GSE179252; Results showed that Low expression of NR0B2 is a risk factor for prognosis(p<0.01)

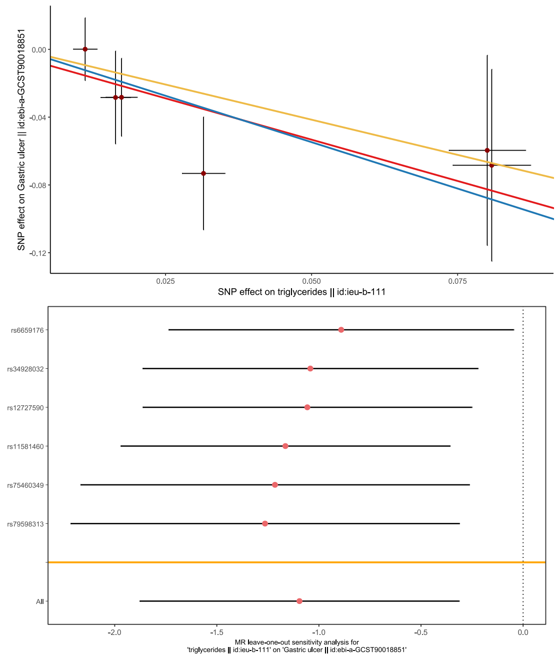

A: Gastric ulcer (ebi-a-GCST90018851)

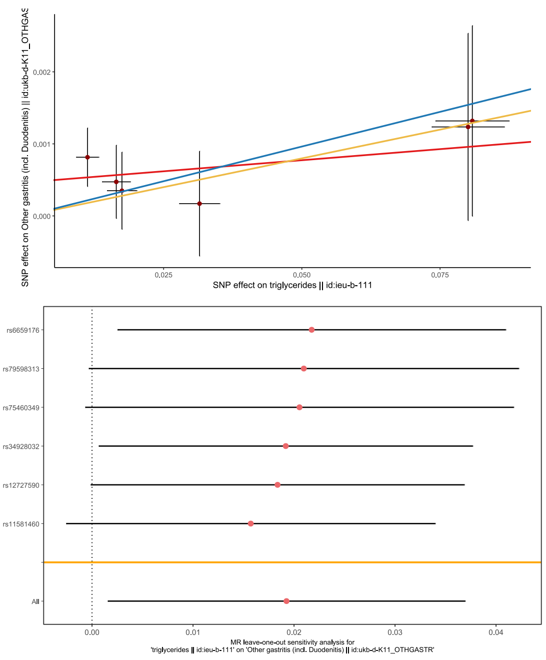

B: Gastritis(ukb- d-K11_OTHGASTR)

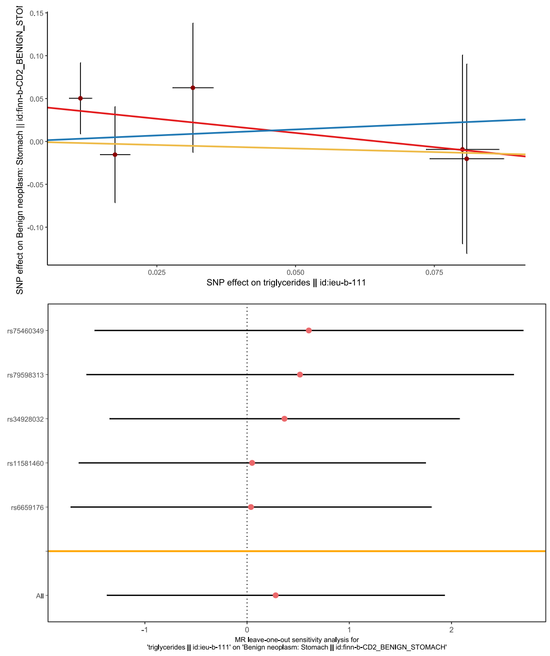

A:finn- b-C3_STOMACH_EXALLC/Malignant neoplasm of stomach

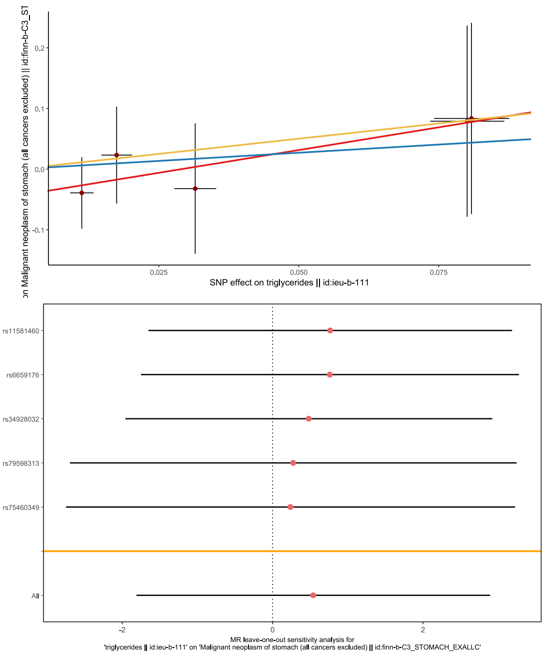

B:finn- b-CD2_BENIGN_STOMACH/Benign neoplasm: Stomach

Figure. S6 The leave-one-out analysis and scatter plot for malignant neoplasm of stomach (finn-b-C3_STOMACH_EXALLC), benign neoplasm: Stomach(finn-b-CD2_BENIGN_STOMACH), gastric ulcer(ebi-a-GCST90018851), and Gastritis(ukb-d-K11_OTHGASTR).

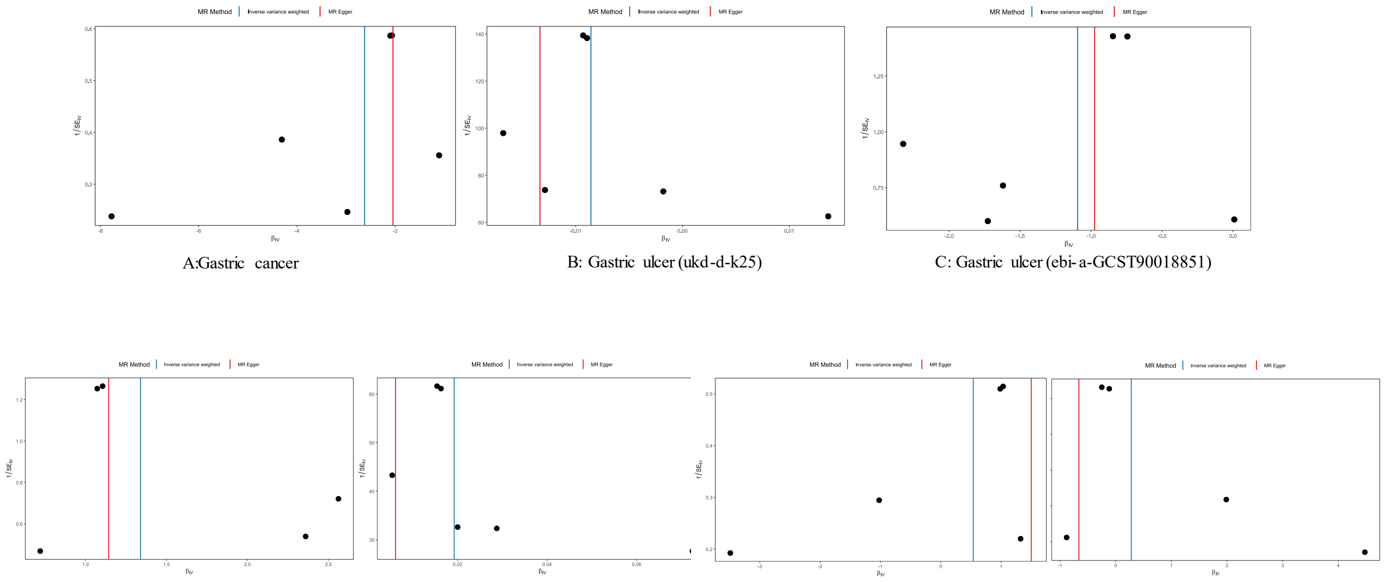

A:Gastric cancer

B: Gastric ulcer (ukd-d-k25)

C: Gastric ulcer (ebi-a-GCST90018851)

D: Gastritis (finn -b-K11_OTHGASTR)

E:G astritis(ukb- d-K11_OTHGASTR)

F:Malignant neoplasm of stomach (finn-b- C3_STOMACH_EXALLC)

G:Benign neoplasm: Stomach (finn-b- CD2_BENIGN_STOMACH)

Figure. S7 Funnel plot for drug Mendelian randomization analyses of the causal effect of the expression of NR0B2 on gastric diseases. horizontal pleiotropy had not been found in positive groups (A, B, C, D and E).

Table. S1 The merged genes by WGCNA

**Subject**
**For GSE138631&GSE26942, GSE17952 and GSE236522**
NR0B2 CA9 KLK11 STARD10 HMGCL ENHO MISP3
**For GSE55696, GSE130823 and GSE233973**
CASP5 CYP1A2 RAVER2 NR0B2 GGN HMOX1 PLCXD2 SLC23A1 IRAK2 MOCS1 FCGBP MMP1 SATB2 ZZEF1 SEMA6C TMEM105 SOX6 SPRR2D LAMA1 ITM2B GPR37L1 MMP24 CDR2L DECR2 OXER1 AQP10 TESK2 GATA5 ASAH2 SIPA1L3 APOC3 DNASE1 CAPNS2 SLC17A4 AQP7P1 DLK2 KRT83 MS4A15 SLFNL1 SPEG TDRG1 THNSL2 UHRF1BP1L MFSD2A C11orf80 SLC52A1 LINC00626
